# Guanine-Based Purines as an Innovative Target to Treat Major Depressive Disorder

**DOI:** 10.3389/fphar.2021.652130

**Published:** 2021-04-13

**Authors:** Roberto F. Almeida, Tiago P. Ferreira, Camila V. C. David, Paulo C. Abreu e Silva, Sulamita A. dos Santos, Ana L. S. Rodrigues, Elaine Elisabetsky

**Affiliations:** ^1^ Departamento de Ciências Biológicas, Programa de Pós-Graduação em Ciências Biológicas, Universidade Federal de Ouro Preto (UFOP), Ouro Preto, Brazil; ^2^ Departamento de Bioquímica, Programa de Pós-Graduação em Ciências Biológicas: Bioquímica, Universidade Federal do Rio Grande do Sul, Porto Alegre, Brazil; ^3^ Departamento de Bioquímica, Centro de Ciências Biológicas, Universidade Federal de Santa Catarina (UFSC), Florianópolis, Brazil

**Keywords:** major depressive disorder, psychopharmacology, purines (source: MeSH), purinergic signaling system, guanine-based purines, guanosine

## Introduction

Major depressive disorder (MDD) is the most prevalent psychiatric disorder worldwide, and the leading disability causes a well-documented syndrome (Liu et al., 2020). MDD treatments are often ineffective, leading to a sizable economic impact onto society and governments (Mauskopf et al., 2009), demanding over 238.3 billion dollars per year in the United States alone (Breslow et al., 2019). Noteworthy, although MDD symptomatology can be found in Hippocratic writings, its pathophysiology remains to be established (Wong and Licinio, 2001). The ability to increase monoamine levels (Rosenblat and McIntyre, 2020) shared by antidepressant agents is the basis for the monoaminergic hypothesis of depression (Hirschfeld, 2000). Although such a neurochemical oriented hypothesis of depression was pioneer and revolutionary in the development of psychopharmacology (Pereira and Hiroaki-Sato, 2018), it has also led to a lack of diversity of strategies in the development of antidepressant agents. As a result until 2009, except for the nonmainstream agomelatine (Norman and Olver, 2019), all antidepressants in the clinic acted by modulating monoaminergic neurotransmission (Berton and Nestler, 2006). Yet 50–60% of the patients do not attain complete remission (Kok and Reynolds, 2017), and respondents require 4–6 weeks for therapeutic effect (Brent, 2016). Developing innovative and fast-acting antidepressants is thus decisive for treating MDD.

The observation of abnormal plasma and cerebrospinal glutamate levels in MDD patients (Machado-Vieira et al., 2009) prompted the suggestion that the glutamatergic system plays a role in the MDD pathogenesis (Scarr et al., 2003; Hashimoto et al., 2007). The hypothesis that modulating the glutamatergic system can be the basis of a new strategy to improve MDD symptomatology was advanced by preclinical models. Various glutamatergic inhibitors exhibit antidepressant-like effect in mice submitted to the forced swim test (FST) (Maj et al., 1992a; Maj et al., 1992b; Moryl et al., 1993; Przegaliński et al., 1997), the tail suspension test (TST) (Trullas and Skolnick, 1990; Layer et al., 1995), and in the chronic stress protocols (Papp and Moryl, 1994; Ossowska et al., 1997; Skolnick et al., 2009). A landmark in this developing line of reasoning was the observation by Berman and collaborators on the rapid and robust antidepressant effect of sub-anesthetic doses of the glutamate NMDA receptor ketamine (Berman et al., 2000), subsequently confirmed by double-blinded clinical trial (Zarate et al., 2006).

Besides the well-documented ketamine mechanism of action in glutamatergic neurotransmission, advances in its pharmacological effect demonstrate that ketamine significantly enriches purinergic metabolism (Weckmann et al., 2017; McGowan et al., 2018). Systemic ketamine increases ATP/ADP and decreases the GTP/GDP ratios in mice hippocampi (Weckmann et al., 2017). A single dose of ketamine administered to mice before contextual fear conditioning-induced depression reveal, by metabolomic analysis, a significantly ATP, AMP, GTP, and GDP increased in the prefrontal cortex, and ADP, AMP, GTP, and GDP boost in the hippocampus, while HYPOX, IMP, and INO levels were found to be decreased in these same structures (McGowan et al., 2018). Changes in purine metabolism were still present after 2 weeks of the ketamine challenge, apparently a pattern for those responsive to ketamine treatment (McGowan et al., 2018). The ketamine incremental effect on nucleotide levels is in line with the demonstration that ketamine enriches the pyrimidine and purine intermediates (Weckmann et al., 2014; McGowan et al., 2018). A possible interpretation is that ketamine can increase the activity of salvage pathways; another is an increase in biosynthesis coupled to a decreased conversion of nucleotides into nucleosides. In any case, increased levels of purine intermediates corroborate the hypothesis raised by Ali-Sisto and colleagues that a hyperactive purine degradation cycle is present in untreated MDD patients (Ali-Sisto et al., 2016).

The pentose phosphate pathway (PPP) is composed by oxidative and non-oxidative phases (Ge et al., 2020); the oxidative phase converts glucose-6-phosphate into ribose-5-phosphate and produces two NADPH molecules (Ge et al., 2020). Ribose-5-phosphate and NADPH are key substrates to protein synthesis, redox balance, and cell integrity (Ge et al., 2020). A single ketamine administration increases mice plasma levels of PPP intermediates (D-ribose-5-phosphate and D-ribulose-5-phosphate), the substrates for purine *de novo* synthesis (McGowan et al., 2018). In agreement with these findings, it has been shown that a single administration of ketamine increased PPP 6-phospho-d-gluconate metabolite in mice hippocampal (Weckmann et al., 2014). Since the metabolites 6-phospho-D-gluconate and D-ribulose-5-phosphate are the result of enzymatic reactions (glucose-6-phosphate dehydrogenase, 6-phosphoglucolactonase, and 6-phosphogluconate dehydrogenase) in a pathway that reduces NADP + to NADPH (Ge et al., 2020), it is plausible to expect that ketamine also increased the NADPH/NADP + ratio. An increased in NADPH/NADP + ratio is in line with the ketamine-induced downstream neuroplasticity-related pathways (e.g., BDNF and mTORC1) (Zanos et al., 2016), protein synthesis, and synaptic plasticity (Zanos et al., 2016; Molero et al., 2018). Since ketamine also modulates purinergic neurotransmission, the ketamine-induced nucleotide and NADPH augmentation might be, at least in part, responsible for the cell proliferation, morphogenesis, and protein synthesis observed after ketamine administration, all of which are relevant for its antidepressant effect.

### Adenine-Based Purines as Antidepressants

Substantial preclinical and clinical data advanced and sustained the involvement of adenosine nucleoside in MDD; see Yamada et al. (2014), López-Cruz et al. (2018), Calker et al. (2019), Bartoli et al. (2020) for reviews. Antidepressant-like effect was obtained by enhancing ATP release from astrocytes, which activated P2X2 receptors in the prefrontal cortex of mice subjected to the social stress depression model (Cao et al., 2013). On the contrary, blocking astrocytic ATP release led to extended depression-like phenotype in the same model (Ren et al., 2018). The relevance of the P2X2 receptor was shown comparing the antidepressant effects of ATP alone and ATP combined with Cu^2+^, a P2X2 receptor enhancer; whereas ATP (4 µM) combined with Cu^2+^ substantially decreased the immobility time in the FST, while ATP (4 µM) alone did not (Cao et al., 2013). Of relevance to antidepressant activity are the data associated with ATP neuroprotection (Jacobson et al., 2012; Ulrich and Illes, 2014; Gampe et al., 2015; Miras-Portugal et al., 2016). ATP can activate GSK3 phosphorylation (at Ser9/21 residues) inhibiting GSK3 activity, thus facilitating neuronal survival and/or function restoration (Jope and Roh, 2006). Ketamine, by affecting purine metabolism and increasing the extracellular nucleotide availability, can activate neuronal and glial nucleotide receptors and regulate intracellular kinases pathways (e.g., PI3K/Akt, GSK3, and ERK1,2) associated with synapto/neurogenesis (Scheuing et al., 2015; Deyama and Duman, 2020). Although these evidences were supported by robust data, several preclinical studies have indicated that the antidepressant effect can also result from P2X7 receptor antagonism (Krügel, 2016; Cheffer et al., 2018). As an immune-modulatory receptor, P2X7 activation is involved with neuroinflammation through microglial activation and interleukin-1β production and also associated with MDD (Krügel, 2016; Cheffer et al., 2018). In fact, the pharmacological inhibition or genetic manipulation of P2X7 has been suggested as a strategy for treating MDD (Iwata et al., 2016; Yue et al., 2017; Farooq et al., 2018; Aricioglu et al., 2019).

In 2005, Calker and Biber (van Calker and Biber, 2005) reported the antidepressant effects of A1 adenosine agonists, and the antidepressant effect of extracellular adenosine signaling was reinforced by others (Hines et al., 2013; Serchov et al., 2015). The enhancement in neuronal A1 receptor expression exerts prophylactic antidepressant effect, while A1 receptor knockout (KO) mice increased depressive-like behavior and were resistant to antidepressant effects of sleep deprivation (Serchov et al., 2015). Additionally, caffeine, a nonselective adenosine receptor antagonist, prevented depressive-like behavior and synaptic changes induced by chronic unpredictable stress (Kaster et al., 2015). Coherent with preclinical observation, important reviews also sustain that caffeine consumption decreases the incidence of depression and suicide risk in patients (Kawachi et al., 1996; Lucas et al., 2014). In the same way, the selective antagonism of A2a adenosine receptors KW6002 or the A2a genetic inactivation mice model of depression seems key to the antidepressant activity (Yacoubi et al., 2001; Kaster et al., 2004; Yamada et al., 2013; Kaster et al., 2015).

### Guanine-Based Purines as Antidepressants

Guanine-based purines, including the nucleotides guanosine 5′-triphosphate (GTP), guanosine 5′-diphosphate (GDP), and guanosine 5′-monophosphate (GMP), the nucleoside guanosine (GUO), and the nucleobase guanine (GUA), have received less attention than classic neurotransmitter as targets in psychiatry. GUO protects against a wide range of deleterious effects in various animal models of neurological disorders (Sopko et al., 2008; Khan et al., 2012). It has been postulated that GTP (as ATP) acts as neurotransmitter (Santos et al., 2006), supporting the idea of a guanine-based purine signaling system (Schmidt et al., 2007). The hypothesis is that various brain insults augment nucleotide release, followed by increased extracellular nucleoside levels, working as part of a restorative arrangement (Pimentel et al., 2013).

The antidepressant-like effect obtained with systemic (i.p.) or central (i.c.v.) GUO in mice models with predictive validity [tail suspension test (TST) and forced swimming test (FST)] was reported in 2012; GUO antidepressant–like activity was blocked by selective inhibitors suggesting the involvement of glutamate NMDA receptors, l-arginine-NO-cGMP, and PI3K-mTOR pathways (Bettio et al., 2012). Prior to this identification of GUO antidepressant-like, it was reported that GUO and guanine derivatives can act as competitive inhibitors of NMDA receptors, prevent NMDA-induced neurotoxicity, and protect against quinolinic acid–induced seizures (Schmidt et al., 2007). Differently than the ketamine modulation in different NMDAR isoforms [selectively inhibition on NMDAR expressed on GABAergic inhibitory interneurons or extra-synaptic GluN2B-containing NMDARs (Zanos and Gould, 2018)], the NMDAR involvement on GUO mechanism of action needs further investigation. Of note, aside for the antidepressant effect, ketamine and GUO share other biological effects, such as amnesic, antinociceptive, and neuroprotective.

Additionally, systemic GUO was also effective in diminishing acute restraint stress-induced depressive-like behavior in the same species (Bettio et al., 2014); biochemical correlates included the attenuation of the stress-induced hippocampal malondialdehyde increase the prevention of changes in the activity of antioxidant enzymes such as glutathione peroxidase (GPx), glutathione reductase (GR), catalase (CAT), and the superoxide dismutase (SOD)/CAT activity ratio (Bettio et al., 2014). Chronic (21 days) orally administered GUO decreased the immobility time in the TST in female mice, positively correlated with increased neuronal differentiation in the ventral (but not dorsal) hippocampal dentate gyrus (Bettio et al., 2016). GUO antidepressant effects were also reported with the combination of subthreshold doses of GUO and ketamine in the novelty-suppressed feeding test (NSFT) (Camargo et al., 2020). Neurochemical analysis showed that 60 min after GUO, there was an increase in mTOR phosphorylation (Ser^2448^) and phospho-p70S6K immunocontent (but no changes in PSD-95, GluA1, and synapsin) in the hippocampus, whereas no changes in phospho-mTOR and phospho-p70S6K were seen in the prefrontal cortex, which presented increased PSD-95, GluA1, and synapsin immunocontent (Camargo et al., 2020). The prefrontal cortex (especially the medial portion), the lateral habenula, and the hippocampus (ventral region) have been consistently implicated in MDD and in antidepressants efficacy (Kupfer et al., 2012; Bettio et al., 2014; Yang et al., 2018).

Using logistic regression, clinical longitudinal studies showed that serum GUO levels are decreased in MDD patients in comparison with healthy controls (Ali-Sisto et al., 2016). Increased uric acid levels were also reported in MDD patients (Kesebir et al., 2014), reinforcing the hypothesis of a hyperactive purine degradation cycle in MDD. Such boosted turnover of nucleotides to nucleosides can be interpreted as an attempt to reestablish the redox homeostasis altered in MDD (Bartoli et al., 2020), congruent with the effect of ketamine on purine metabolism (Weckmann et al., 2017; McGowan et al., 2018). Reinforcing that PI3K/Akt/mTOR is required for GUO antidepressant–like effects, Rosa and colleagues (Rosa et al., 2019) reported that sub-effective doses of GUO combined with GSK-3β inhibitors reduced immobility at the TST, a result compatible with the PI3k/Akt ability to inhibit GSK-3β signaling. To explain the increased β-catenin content found at the hippocampus and prefrontal cortex cell nuclear fractions, the same authors suggested that GSK-3β is inhibited by GUO, resulting in cytosol β-catenin accumulation and subsequent translocated into the nucleus (Rosa et al., 2019). GUO antidepressant–like effects were blocked by MEK1/2 inhibitors, suggesting that GUO can also activate the MAPK/ERK pathway, further reinforcing the involvement of the mTOR signaling in GUO effects. GUO antidepressant–like effects were abolished by the co-administration of GUO and HO-1 inhibitors, while systemic GUO increased the nuclear factor Nrf-2 in the hippocampus and prefrontal cortex (Rosa et al., 2019) observations compatible with the known MAPK/ERK and/or GSK-3β/PI3K/Akt activation of Nrf2.

The bilateral olfactory bulbectomy (OBX) is considered as the best suited rodent model to investigate novel fast-onset antidepressants (Ramaker and Dulawa, 2017). We established that a single intraperitoneal injection of GUO (7.5 mg/kg) reversed the OBX-induced anhedonia-like behavior and recognition memory impairment in mice (Almeida et al., 2020). As the effects of GUO and ketamine were comparable at OBX and both abolished by rapamycin, the study provided additional evidence for the requirement of the mTOR pathway in GUO and ketamine mechanism of action as antidepressant agents (Almeida et al., 2020).

Ketamine antidepressant effects apparently require the activation of molecular targets downstream to mTOR (primarily the protein kinase p70S6K) (Duman et al., 2012; Fraga et al., 2020), ultimately facilitating protein translation, cell growth, proliferation, formation, maturation, and function of new spine synapses (Zito et al., 2009). Considering that (Liu et al., 2020), GUO and ketamine show fast-onset antidepressant-like effect requiring the mTOR pathway (Bettio et al., 2012; Mauskopf et al., 2009). GUO and ketamine modify purine metabolism (Almeida et al., 2017; McGowan et al., 2018) and (Breslow et al., 2019) that the *in vitro* (Su et al., 2013) and *in vivo* (Bettio et al., 2012) GUO neurotrophic and neuritogenic effects involve the same pathways reported for ketamine; it is tempting to speculate that scrutiny of neurochemical correlates of compounds that present fast-onset antidepressant effects might reveal a common set of molecular targets (Weckmann et al., 2017; McGowan et al., 2018).

Although ketamine opened a whole new avenue and hope for a more efficacious management of MDD, other compounds with fast-onset antidepressant agents did not come forward. Exploratory studies support the potential of GUO as a fast-onset antidepressant, with a safe profile (Molz et al., 2011; Tasca et al., 2018). The use of ketamine is limited by its adverse profile, including psychomotor and addictive effects (Lener et al., 2017). On the contrary, compelling evidence shows that GUO is safe, well tolerated, and not associated with major side effects (Molz et al., 2011; Tasca et al., 2018), which increase the chance of well tolerability in long-term treatments. The commonalities of ketamine and GUO mechanisms of action suggest that a better understanding on the role of guanine-based purines in MDD is relevant and necessary for innovation in the field.
